# Gout flare burden as a marker of chronic kidney disease risk

**DOI:** 10.1186/s12882-026-04904-2

**Published:** 2026-03-17

**Authors:** Hung-Ping Wang, Chien-Hung Lin, Peir-Haur Hung, Chun Lee, Solomon Chih-Cheng Chen

**Affiliations:** 1 Department of Internal Medicine, Ditmanson Medical Foundation Chia- Yi Christian Hospital, Chiayi City, 600566 Taiwan; 2 Department of Pediatrics, Taipei Veterans General Hospital, Taipei, 112201 Taiwan; 3 School of Medicine, National Yang Ming Chiao Tung University, Taipei, 112304 Taiwan; 4 College of Science and Engineering, Fu Jen Catholic University, New Taipei, 242062 Taiwan; 5 Clinical Data Center, Ditmanson Medical Foundation Chia-Yi Christian Hospital, Chiayi city, Taiwan; 6 Department of Pediatrics, Ditmanson Medical Foundation Chia-Yi Christian Hospital, Zhongxiao Rd., East Dist, Chiayi City, 600566 Taiwan; 7 Department of Pediatrics, College of Medicine, Taipei Medical University, Taipei, 110301 Taiwan

**Keywords:** Hyperuricemia, Inflammation, Renal insufficiency, Urate-lowering therapy, Electronic health records, Retrospective cohort study

## Abstract

**Background:**

Gout is a common inflammatory arthritis with substantial cardiometabolic and renal comorbidity. Although hyperuricemia and baseline kidney function are established predictors of chronic kidney disease (CKD), the prognostic value of long-term gout flare frequency is unclear. This study aimed to examine the association between annual gout flare frequency and the risk of incident CKD.

**Methods:**

We conducted a large-scale retrospective cohort study using de-identified electronic health record data from the TriNetX Global Collaborative Research Network. Adults with at least one treated gout flare in 2017 and preserved baseline renal function (eGFR ≥ 60 mL/min/1.73 m²) were included. Exposure was defined by the annual frequency of treated gout flares. The primary outcome was incident CKD stage 3–5 or end-stage renal disease (ESRD). To minimize reverse causation, a 180-day lag period was applied before the start of follow-up. Multivariable Cox proportional hazards models were used to estimate adjusted hazard ratios (aHRs) for incident CKD over a 7-year follow-up period, adjusting for age, sex, comorbidities (hypertension, diabetes, heart disease, obesity), and urate-lowering therapy use.

**Results:**

Among 29,321 eligible adults, a higher annual gout flare frequency was independently associated with an increased risk of incident CKD in a graded, dose-dependent manner. Compared with individuals experiencing one flare per year, the risk of incident CKD was significantly higher among those with ≥ 5 flares per year, with aHRs ranging from approximately 1.20 to 1.46 across follow-up years. In contrast, experiencing two flares per year did not confer a statistically significant increase in risk relative to the reference group. Risk separation was evident by year 3 and persisted throughout the 7-year observation period.

**Conclusions:**

Recurrent gout flares are associated with a progressively increased risk of incident CKD, suggesting that flare frequency serves as a pragmatic, patient-centered prognostic marker for long-term renal risk assessment. These findings underscore the importance of optimizing flare prevention and urate-lowering strategies in high-risk populations to potentially mitigate adverse renal outcomes.

## Introduction

Gout is a common inflammatory arthritis characterized by recurrent acute flares, chronic low-grade systemic inflammation, and important cardiometabolic and renal consequences [1–4]. Patients with gout have a higher prevalence of chronic kidney disease (CKD) and an increased risk of adverse renal outcomes than the general population, reflecting a complex interplay of hyperuricemia, shared comorbidities, and treatment-related factors [3–6].

While hyperuricemia and baseline renal function are established determinants of CKD risk in gout [7, 8], the prognostic relevance of gout flare frequency for renal outcomes remains poorly defined. Acute flares represent periods of heightened systemic inflammation and increased healthcare utilization, and higher flare frequency is associated with greater disease burden and patient concern [9, 10]. Recurrent inflammatory stress during flares has been linked to progressive organ injury, including a transiently increased risk of myocardial infarction and stroke [4, 11], yet whether sustained flare burden similarly signals an increased long-term risk of CKD remains unclear.

Flare counts are simple, patient-reportable, and actionable at the point of care, and flare frequency is an intuitive indicator of disease control from the patient perspective [1, 9, 10, 12]. In contrast, serum urate—the standard biochemical target in gout management—fluctuates over time, and longitudinal monitoring is often inconsistent, particularly in primary care [13–16]. As a result, most prior studies have used static serum urate measurements or a binary diagnosis of gout when assessing renal risk, rather than treating flare burden as a dynamic exposure. Annual gout flare frequency therefore represents a pragmatic measure of longitudinal disease activity whose association with long-term renal outcomes remains insufficiently characterized.

Using data from the TriNetX Global Collaborative Research Network [17], we conducted a large retrospective cohort study to examine the association between annual gout flare frequency and the risk of incident CKD. We hypothesized that flare frequency functions as a dynamic, patient-centered prognostic marker for CKD risk, with a graded increase in hazard across flare categories after multivariable adjustment.

## Methods

### Study design and data source

This retrospective cohort study utilized de-identified electronic health record (EHR) data from the TriNetX Global Collaborative Research Network, a large multinational federated network comprising data from healthcare organizations across multiple countries. Available data elements include demographics, diagnoses coded using the International Classification of Diseases, Tenth Revision (ICD-10), procedures coded using Current Procedural Terminology (CPT), and prescription records mapped to RxNorm.

### Study population

Adults aged ≥ 18 years with at least one treated gout flare documented between January 1 and December 31, 2017, were eligible for inclusion. To ensure preserved baseline kidney function, patients were required to have a baseline estimated glomerular filtration rate (eGFR) ≥ 60 mL/min/1.73 m² prior to the index date.

Patients were excluded if they had any prior diagnosis of CKD stage ≥ 3, end-stage renal disease (ESRD), dialysis, kidney transplantation, or acute kidney injury at any time before cohort entry, as well as those with missing baseline eGFR data.

The index date was defined as the date of the first treated gout flare in 2017. Follow-up began 180 days after the index date to reduce reverse causation and continued until the earliest occurrence of incident CKD, death, loss to follow-up, or a maximum of 7 years.

### Exposure: annual gout flare frequency

Annual gout flare frequency was defined based on healthcare encounters coded for gout (ICD-10 code M10.xx) accompanied by flare-related anti-inflammatory treatment, including nonsteroidal anti-inflammatory drugs (NSAIDs), colchicine, or systemic corticosteroids, within a predefined time window.

Patients were categorized according to their annual flare frequency into four groups: 1–2, 3–4, 5–6, and ≥ 7 flares per year. For baseline descriptive comparisons (Table 1), categories were collapsed into low (1–2), moderate (3–4), and high (≥ 5) flare burden to improve interpretability, whereas time-to-event models and figures used the full flare-frequency categories as specified.

**Table 1:** Baseline demographic and clinical characteristics of patients stratified by annual gout flare frequency

Characteristics	Total *n* = 29,321	Low (1–2 flares) *n* = 15,032	Moderate (3–4 flares) *n* = 5,820	High (≥ 5 flares) *n* = 8,469
Age, years (mean ± SD)	70 ± 13	71 ± 13	70 ± 12	68 ± 12
Sex, n (%)				
Male	23,532 (80.3)	11,710 (77.9)	4,710 (80.9)	7,112 (84.0)
Female	5,789 (19.7)	3,322 (22.1)	1,110 (19.1)	1,357 (16.0)
Comorbidities, n (%)				
Hypertension (I10-I15)	25,564 (87.2)	12,871 (85.6)	5,192 (89.2)	7,501 (88.6)
Diabetes mellitus (E08-E13)	13,208 (45.1)	6,548 (43.6)	2,633 (45.2)	4,027 (47.5)
Ischemic heart disease (I20-I25)	11,873 (40.5)	6,001 (39.9)	2,406 (41.3)	3,466 (40.9)
Obesity (E65-E68)	15,008 (51.2)	7,181 (47.8)	3,156 (54.2)	4,671 (55.2)
ULT use within 12 months before/after index, n (%)				
Before	6,284 (21.4)	3,423 (22.8)	1,217 (20.9)	1,644 (19.4)
After	6,515 (22.2)	2,393 (15.9)	1,365 (23.5)	2,757 (32.6)

Note: Values are presented as mean ± standard deviation (SD) for continuous variables and n (%) for categorical variables. Flare frequency was categorized as Low (1–2 flares/year), Moderate (3–4 flares/year), and High (≥ 5 flares/year). Urate-lowering therapy (ULT) use was stratified by time relative to the index event, defined as ULT use within 12 months prior to or within 12 months following the index date. Chi-square tests were used to compare categorical variables across groups. Across flare frequency categories, pre-index urate-lowering therapy use showed a decreasing trend (*p* = 0.040), whereas post-index use showed an increasing trend (*p* = 0.035), as assessed by the Cochran–Armitage trend test

### Outcome: incident chronic kidney disease

The primary outcome was incident CKD stage 3–5 or ESRD, identified using ICD-10 diagnostic codes (N18.3–N18.6 and Z99.2). This administrative definition was used to capture clinically recognized CKD events in a large real-world cohort. To further minimize reverse causation, CKD events occurring within the first 180 days after the index date were excluded from outcome ascertainment.

### Covariates

Prespecified covariates included age at index date, sex, hypertension (ICD-10 I10–I15), diabetes mellitus (E08–E13), ischemic heart disease (I20–I25), obesity (E65–E68), and baseline ULT use. All covariates were assessed prior to or at the index date and selected a priori based on clinical relevance and existing literature.

### Statistical analysis

Baseline characteristics were summarized using descriptive statistics and compared across flare frequency groups (Table 1). Trends across increasing flare frequency categories for categorical variables were assessed using the Cochran–Armitage trend test. Cumulative incidence of CKD was estimated using Kaplan–Meier methods and assessed at 3, 5, and 7 years of follow-up, with censoring at the last available follow-up (Fig. 2).

Multivariable Cox proportional hazards models were used to evaluate the association between annual gout flare frequency and incident CKD, adjusting for prespecified covariates. Adjusted hazard ratios (aHRs) with 95% confidence intervals (CIs) were reported (Fig. 3; Table 3).

Time-specific sensitivity analyses were conducted by repeating Cox models at each follow-up year from years 2 through 7 to assess the temporal stability of associations. Subgroup analyses were performed stratified by age, sex, and ULT use. All analyses were conducted within the TriNetX Analytics platform using standard survival modeling tools. Statistical significance was defined as a two-sided *p* value < 0.05.

## Results

### Study population

**Fig. 1:**
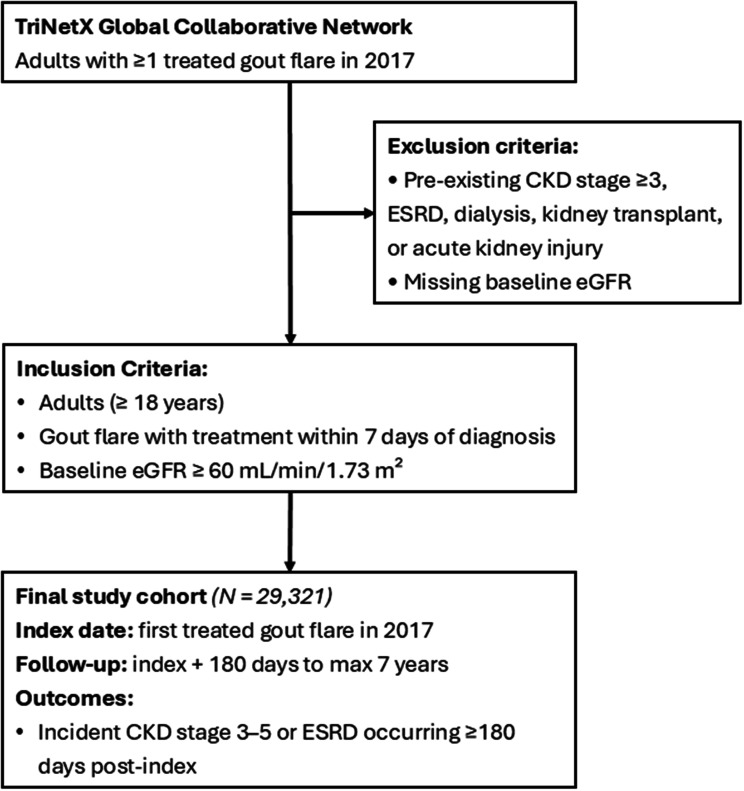
Flow diagram of cohort selection. Flow diagram of cohort selection from the TriNetX Global Collaborative Research Network. Adults with at least one treated gout flare in 2017 were identified, and predefined exclusion criteria—including pre-existing advanced CKD and missing baseline kidney function—were applied to derive the final analytic cohort with preserved baseline renal function (eGFR ≥ 60 mL/min/1.73 m²). Follow-up began 180 days after the index flare and continued for up to 7 years for ascertainment of incident CKD

A total of 29,321 adults with at least one treated gout flare in 2017 met the inclusion criteria and were included in the final analysis (Fig. 1). Follow-up commenced 180 days after the index flare and continued for up to 7 years for ascertainment of incident CKD.

### Baseline characteristics by flare frequency

Baseline demographic and clinical characteristics stratified by annual gout flare frequency are shown in Table 1. Patients with higher flare frequency tended to be younger and more frequently male. The prevalence of cardiometabolic comorbidities was high across all flare frequency groups, with modest differences observed between categories.

ULT use demonstrated a distinct temporal pattern relative to the index flare. Pre-index ULT use (within 12 months before the index event) was similar across flare frequency groups (approximately 20%). In contrast, post-index ULT use (within 12 months after the index event) increased progressively with flare burden, with the highest use observed among patients with ≥ 5 flares per year (Table 1).

### Cumulative Incidence and absolute risk of CKD

**Fig. 2:**
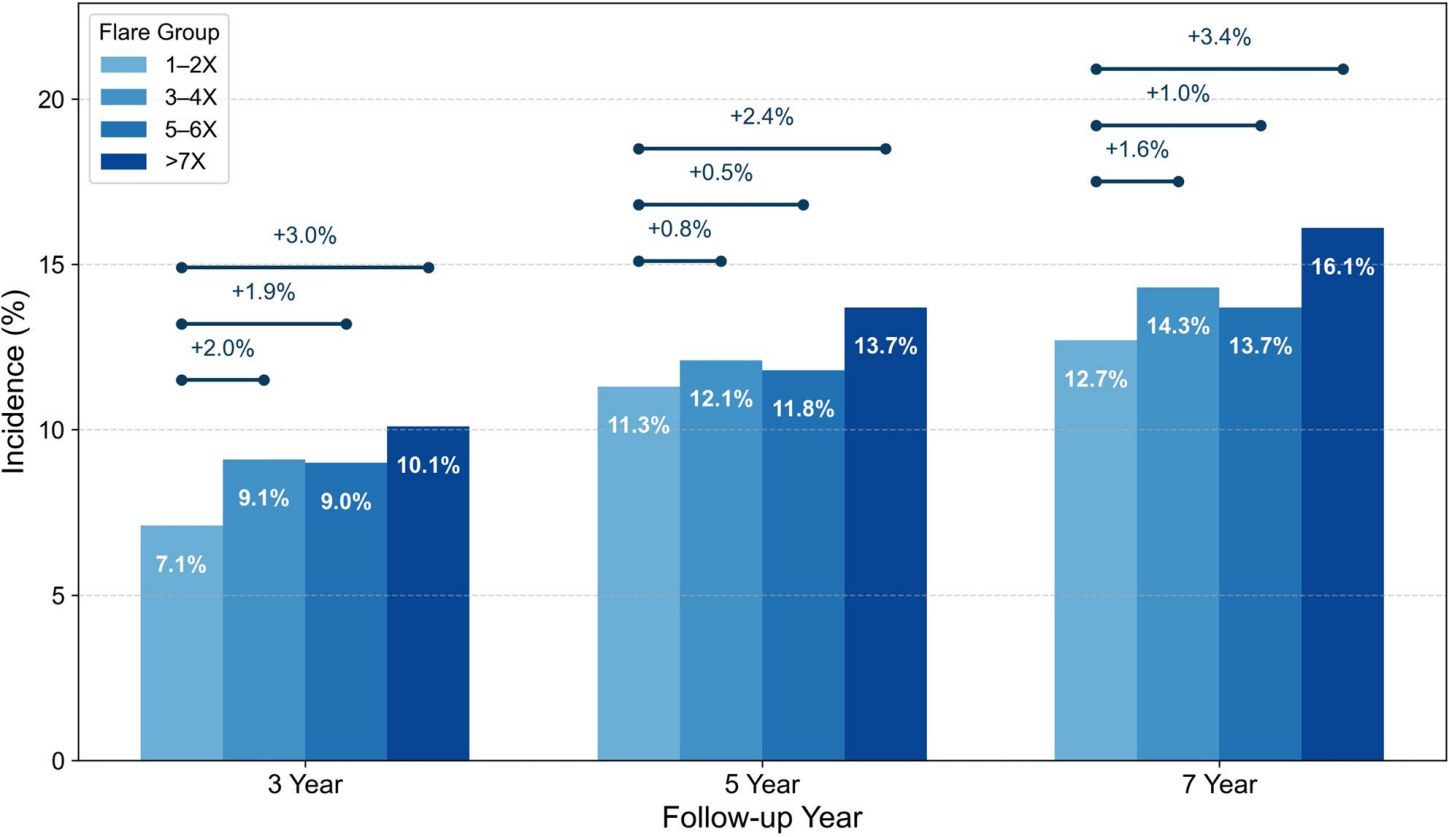
Cumulative incidence and absolute risk increase of CKD by flare frequency. Kaplan–Meier–estimated cumulative incidence of incident CKD at 3, 5, and 7 years, stratified by annual gout flare frequency. Bars represent cumulative incidence within each flare group, and annotations indicate absolute risk increases compared with the 1–2 flare group at each time point

The cumulative incidence of incident CKD increased with higher annual gout flare frequency over time (Fig. 2). Risk separation between flare groups was evident by 3 years of follow-up and persisted through 7 years. Patients with ≥ 7 flares per year consistently exhibited the highest cumulative incidence, whereas those with 1–2 flares per year had the lowest risk. The absolute risk difference between flare groups widened over time, indicating a flare-dependent gradient in CKD risk (Fig. 2).

### Association between flare frequency and incident CKD

**Fig. 3:**
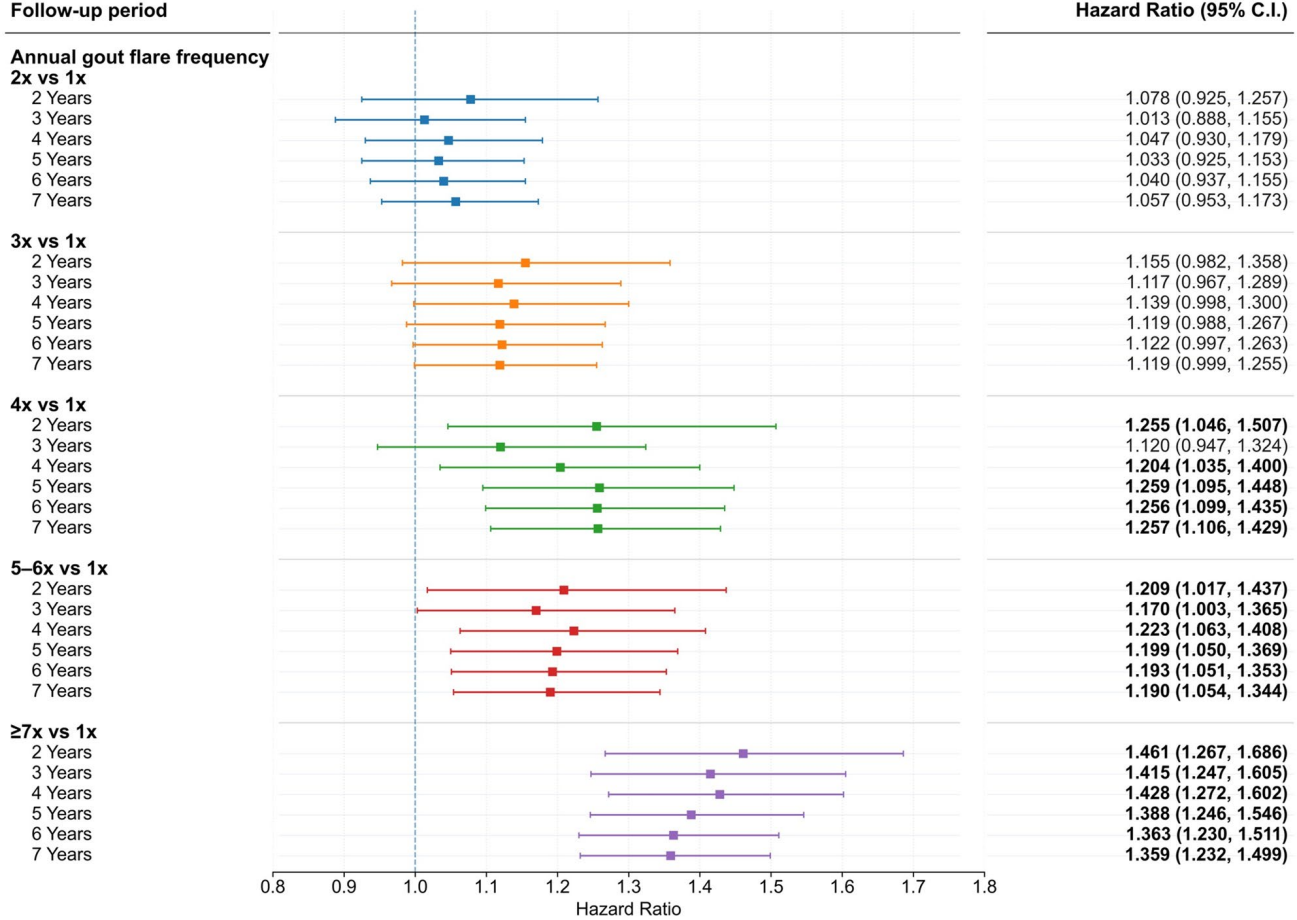
Adjusted hazard ratios for incident CKD by annual gout flare frequency. Adjusted hazard ratios and 95% confidence intervals for incident CKD across annual gout flare frequency categories over 7 years of follow-up, with 1 flare per year as the reference group. Models were adjusted for age, sex, hypertension, diabetes mellitus, ischemic heart disease, overweight/obesity, and baseline urate-lowering therapy use

Adjusted hazard ratios for incident CKD according to annual gout flare frequency are presented in Fig. 3. For clarity, cumulative incidence analyses used patients with 1–2 flares per year as the low-risk reference group (Fig. 2), whereas hazard ratio models used 1 flare per year as the statistical reference by design, reflecting clinical risk stratification and model stability considerations. Compared with patients experiencing 1 flare per year, those with higher flare burdens demonstrated progressively increased CKD risk. This association was most pronounced and consistent among patients with ≥ 5 flares per year, with adjusted hazard ratios ranging approximately from 1.20 to 1.46 across follow-up years. In contrast, patients with 2 flares per year did not show a statistically significant increase in CKD risk relative to the reference group (Fig. 3).

### Covariate effects and clinical risk stratification

At 5 years of follow-up, multivariable analyses demonstrated that higher flare frequency remained independently associated with increased CKD risk after adjustment for age, sex, hypertension, diabetes mellitus, ischemic heart disease, overweight/obesity, and ULT (Table 2). Traditional cardiometabolic comorbidities, particularly hypertension and diabetes mellitus, were also strongly associated with CKD risk across all flare strata.

**Table 2:** Covariate-specific adjusted hazard ratios for incident CKD at 5 years, stratified by annual gout flare frequency

Covariate	2x vs. 1x HR (95% C.I.)	3x vs. 1x HR (95% C.I.)	4x vs. 1x HR (95% C.I.)	5-6x vs. 1x HR (95% C.I.)	≥ 7x vs. 1x HR (95% C.I.)
Flare frequency (vs. 1x)	1.033 (0.925, 1.153)	1.119 (0.988, 1.267)	**1.259 (1.095**,** 1.448)**	**1.199 (1.050**,** 1.369)**	**1.388 (1.246**,** 1.546)**
Male	0.969 (0.859, 1.093)	0.952 (0.838, 1.081)	0.947 (0.829, 1.081)	0.912 (0.801, 1.040)	0.919 (0.818, 1.033)
Age at Index	**1.056 (1.050**,** 1.062)**	**1.055 (1.049**,** 1.061)**	**1.055 (1.049**,** 1.061)**	**1.055 (1.049**,** 1.061)**	**1.053 (1.048**,** 1.059)**
Type 2 diabetes mellitus	**1.665 (1.494**,** 1.855)**	**1.594 (1.421**,** 1.787)**	**1.636 (1.451**,** 1.845)**	**1.668 (1.483**,** 1.877)**	**1.576 (1.421**,** 1.748)**
Ischemic heart diseases	**1.525 (1.364**,** 1.704)**	**1.605 (1.428**,** 1.805)**	**1.609 (1.423**,** 1.819)**	**1.565 (1.387**,** 1.765)**	**1.699 (1.528**,** 1.890)**
Overweight and obesity	**1.456 (1.303**,** 1.628)**	**1.418 (1.259**,** 1.598)**	**1.432 (1.265**,** 1.623)**	**1.346 (1.191**,** 1.521)**	**1.543 (1.388**,** 1.715)**
Hypertensive diseases	**1.863 (1.534**,** 2.261)**	**1.844 (1.504**,** 2.262)**	**1.821 (1.478**,** 2.245)**	**1.734 (1.418**,** 2.119)**	**1.767 (1.473**,** 2.119)**
Using ULT medicines	**1.428 (1.276**,** 1.598)**	**1.483 (1.315**,** 1.673)**	**1.452 (1.282**,** 1.645)**	**1.410 (1.246**,** 1.596)**	**1.550 (1.378**,** 1.743)**

Note: Hazard ratios (HRs) and 95% confidence intervals (CIs) were derived from multivariable Cox proportional hazards models evaluating incident CKD at 5 years. Results are stratified by annual gout flare frequency, with patients experiencing 1 flare per year serving as the reference group. All models were adjusted for age, sex, hypertension, diabetes mellitus, ischemic heart disease, overweight/obesity, and urate-lowering therapy at baseline

Based on these findings, a clinical CKD risk stratification framework anchored to gout flare burden is summarized in Table 3, highlighting increasing renal risk signals with escalating flare frequency.

**Table 3:** Clinical CKD risk stratification framework by gout flare burden

Annual flare burden	Relative CKD risk (association vs. 1 flare)	Supporting evidence (aHR range, years 4–7)	Recommended clinical actions
Low (1–2 flares)	Baseline risk	aHR ~ 1.0	Standard gout and comorbidity management per guidelines
Moderate (3–4 flares)	Elevated risk (association)	aHR 1.12–1.26	Proactive renal monitoring (annual eGFR and UACR) may be warranted; reinforce ULT adherence and lifestyle measures
High (≥ 5 flares)	Higher and consistent risk (association)	aHR 1.20–1.46	Consider intensifying ULT to achieve flare reduction; review adherence; consider nephrology referral for comanagement

Note: This framework is derived from adjusted hazard ratios in the present study (see Fig. 3) and is intended as a clinical translation tool to highlight potential risk signals rather than to define treatment thresholds or clinical guidelines. Relative risk is expressed versus 1 flare per year (model reference); clinically, ≤ 2 flares per year represents a low-flare range with no measurable excess risk in this cohort

## Discussion

In this large-scale retrospective analysis using data from the TriNetX Global Collaborative Research Network, our findings identify annual gout flare frequency as a pragmatic prognostic indicator for incident CKD. Because it can be easily assessed in routine care and reflects patients’ lived experience of flares, it serves as a patient-centered marker of long-term renal risk. An increasing annual flare burden was associated with a progressively higher risk of CKD over a 7-year follow-up period, showing a graded, dose–response pattern. This association remained robust after adjustment for demographic characteristics, cardiometabolic comorbidities, and ULT use. Patients with frequent flares (≥ 5 per year—and most notably those with ≥ 7) had the greatest CKD risk, whereas those with a low flare burden (1–2 flares per year) showed no statistically significant increase in risk compared with the reference group.

These findings expand upon the established association between gout and CKD—a relationship traditionally linked to hyperuricemia, impaired renal urate excretion, and shared metabolic risk factors [2, 15, 16]. While prior research has largely focused on baseline renal status or static biochemical markers, our analysis underscores gout flare frequency as a pragmatic clinical indicator that reflects longitudinal disease activity and cumulative inflammatory burden. Given that acute flares are marked by intense systemic inflammation, recurrent episodes are associated with a significantly higher risk of progressive renal impairment over time [6, 14]. In this context, flare frequency serves as a clinical complement to serum urate, signaling disease activity that sporadic laboratory measurements may overlook—especially in real-world settings where urate monitoring is often irregular and insufficient for longitudinal tracking.

Notably, the lack of measurable excess CKD risk among patients experiencing 1–2 flares per year—in contrast to the increased risk observed at ≥ 3 flares—suggests a potential clinical threshold effect [9]. Infrequent flares may represent a relatively stable clinical phenotype even among individuals with established gout, supporting the designation of the low-flare group as a clinically relevant reference category for risk assessment. Collectively, these observations suggest that signals of heightened renal risk may emerge primarily when flares become recurrent rather than remaining sporadic, underscoring the clinical value of tracking repeated flare activity as an indicator of renal vulnerability.

Another key observation was the temporal relationship between gout flare burden and the use of ULT. Our analysis revealed a subtle but significant decreasing trend in pre-index ULT exposure as flare frequency increased (*p* = 0.040; Table 1), whereas post-index initiation and intensification increased progressively with higher flare burden (*p* = 0.035; Table 1). This observed pattern suggests that ULT escalation in this cohort often followed, rather than preceded, the accumulation of flares. Consequently, patients experiencing more frequent flares appeared more likely to receive intensified therapy only after disease activity had already escalated [8]—a pattern that may reflect a predominantly reactive, rather than proactive, approach to flare prevention in real-world clinical practice.

Beyond direct inflammatory stress, several intermediate pathways may link a high flare burden to incident CKD. First, recurrent flares are associated with potential renal injury through cardiorenal mechanisms. Recent evidence has highlighted a significant, transiently increased risk of acute myocardial infarction and stroke in the period immediately following a gout flare [4, 11]. The systemic inflammatory response and hemodynamic stress associated with these events may lead to complications such as new-onset heart failure or reduced renal perfusion, which are associated with accelerated renal injury.

Second, the pharmacological management of frequent flares—specifically the repeated use of NSAIDs—represents a plausible mediator of renal risk. The nephrotoxic effects of NSAIDs, including impaired renal autoregulation, reduced perfusion, and the promotion of chronic interstitial injury, are well-recognized, particularly in patients with existing cardiometabolic comorbidities. A higher annual flare burden is often associated with more frequent NSAID exposure, which potentially increases the cumulative risk of CKD progression. Consequently, reducing flare frequency may confer dual benefits by mitigating the systemic inflammatory burden and limiting episodic exposure to potentially nephrotoxic therapies.

From a clinical perspective, our findings suggest that annual gout flare frequency may serve as a pragmatic and readily accessible risk stratification tool for identifying individuals at an increased risk of CKD. As flare burden escalates, so does the impact of gout on patients’ lives, particularly regarding illness perceptions and concerns over long-term consequences [10]. In contrast to serum urate levels—which fluctuate over time and often lack consistent longitudinal monitoring in routine practice—flare frequency is easily obtainable during clinical encounters. When combined, flare history and biochemical data offer complementary perspectives on gout control, analogous to the integrated use of clinical patterns and laboratory markers in other chronic disease models. Based on these observations, patients with recurrent flares may benefit from heightened renal surveillance, earlier optimization of ULT, and more rigorous management of cardiometabolic comorbidities [4]. The proposed risk stratification framework (Table 3) is intended to highlight these prognostic signals to guide clinical vigilance rather than to define rigid prescriptive treatment thresholds.

The strengths of this study include its large-scale sample size, extended follow-up duration, and the use of real-world data leveraging a multinational healthcare network. The flare-anchored design facilitated the evaluation of both absolute and relative CKD risks over a multi-year horizon. However, several limitations warrant consideration. First, the observational nature of the study precludes direct causal inference. Second, because gout flares and incident CKD were identified using ICD-10 diagnostic codes, there is a potential for under-ascertainment, particularly concerning subclinical or early-stage disease. While laboratory-based eGFR values were utilized to ensure preserved kidney function at baseline, our outcome definition focuses on clinically recognized CKD requiring medical attention; consequently, our findings likely represent conservative risk estimates. Third, residual confounding from unmeasured factors—such as socioeconomic status, diet, alcohol intake, and medication adherence—cannot be entirely excluded given the inherent constraints of electronic health record data. Fourth, our operational definition of gout flares required documented healthcare encounters and specific prescriptions, which prioritized the capture of clinically significant flares but may have omitted milder, self-managed episodes. Finally, ULT exposure was analyzed as a binary variable due to the lack of granular data on dosing intensity, persistence, or compliance. Furthermore, while follow-up extended to 7 years, the cumulative incidence of CKD plateaued after the first 5 years; therefore, later-term estimates influenced by potential competing risks and attrition should be interpreted with descriptive caution.

## Conclusions

In conclusion, a higher annual gout flare frequency is independently associated with an increased risk of incident CKD in a graded, dose-response manner. These findings support the observation that recurrent gout flares signal heightened renal vulnerability and underscore the role of flare burden as a clinically accessible, patient-centered marker for long-term renal risk assessment. Identifying this high-risk population presents a critical opportunity for the proactive optimization of urate-lowering strategies to potentially mitigate adverse renal outcomes in patients with gout.

## Data Availability

This was a retrospective cohort study using de-identified data from the TriNetX global research network. Individual participant data will not be shared. Access to the TriNetX platform is restricted to participating institutions and cannot be transferred externally. No additional study-related documents will be made available.
